# Ripening of bananas using *Bowdichia virgilioides* Kunth leaves

**DOI:** 10.1038/s41598-019-40053-3

**Published:** 2019-03-05

**Authors:** Rivaildo da Costa Nascimento, Oliveiros de Oliveira Freire, Lylian Souto Ribeiro, Mikael Bolke Araújo, Fernando Luiz Finger, Marcus Alvarenga Soares, Carlos Frederico Wilcken, José Cola Zanuncio, Wellington Souto Ribeiro

**Affiliations:** 1 Departamento de Agroecologia e Agropecuária, Sítio Imbaúba s/no, Campus II, Universidade Estadual da Paraíba, 58117-000 Lagoa Seca, Paraíba Brazil; 2 Departamento de Fitotecnia de Ciências Ambientais, Campus II, Universidade Federal da Paraíba, 58397-000 Areia, Paraíba Brazil; 3 Departamento de Fitossanidade, Faculdade de Agronomia Eliseu Maciel, Universidade Federal de Pelotas, 96010-610 Capão do Leão, Rio Grande do Sul Brazil; 4 Departamento de Fitotecnia, Universidade Federal de Viçosa, 36570-900 Viçosa, Minas Gerais Brazil; 5 Departamento de Agronomia, Universidade Federal do Vale do Jequitinhonha e Mucuri, 39803-371 Diamantina, Minas Gerais Brazil; 6 Departamento de Proteção Vegetal, Universidade Estadual Paulista, 18610-307 Botucatu, Brazil; 7 Departamento de Entomologia/BIOAGRO, Universidade Federal de Viçosa, 36570-000 Viçosa, Minas Gerais Brazil; 8 Programa de Pós-graduação em Horticultura Tropical, Universidade Federal de Campina Grande, 8, Rua Jairo Vieira Feitosa, 58840-000 Pombal, Paraíba Brazil

## Abstract

Bananas are usually ripened with calcium carbide (CaC_2_), a dangerous substance that can cause food poisoning. The objective was to test the empirical ripening banana method using *Bowdichia virgilioides* leaves compared to carbide. Ripening tests were carried out using ‘Pacovan’ banana fruits with *B. virgilioides* leaves and carbide following the empirical method used by Borborema farmers, Paraíba, Brazil. *Bowdichia virgilioides* leaves induced increased respiration and ascorbic acid production and reduced acidity, chlorophyll and pH in banana fruits like CaC_2_. Leaves of *B. virgilioides* induce ripening of ‘Pacovan’ banana with safer and same results than with CaC_2_.

## Introduction

*Musa* sp. has global economic importance as one of the most important basic food sources along with rice, maize and wheat^1^. About 150 countries produce this fruit, totaling more than 100,000 t year^−1^. India (25,000 t year^−1^), China (10,000 t year^−1^), Philippines (8,900 t year^−1^), Ecuador (6,770 t year^−1^) and Brazil are the main banana producers in the world^2–4^. These countries have socioeconomic similarities highlighting the importance of this culture for the economy and regional development^5^, mainly for small producers^6^. Simplified cultivation processes, high demand and acceptance of bananas in the domestic market of these countries, enable their production, albeit often with low quality and/or productivity^7^. In addition, cooperatives and associations are important channels to organize and support banana farming activities^8^.

Banana fruit ripening depends on intrinsic factors such as respiration and ethylene production/sensitivity^9^ and market requirements^10^. Locally marketed bananas may be harvested at a later maturation stages, but bananas for export should be harvested the day before or the day of shipment^11^. In this case, maturation standardization is induced by air conditioning^12^ to plan banana commercialization and industrialization^13^. Acetylene and traces of this compound, produced by calcium carbide (CaC_2_), accelerate and standardize ripening (color uniformity) without losses to quality or taste^14^. These products cannot be used in organic or agroecological production systems^15^, but have no restrictions in countries such as Bangladesh, India, Nepal and Pakistan^16^.

CaC_2_ can cause adverse effects to human health, such as choking, motor coordination problems, headaches, respiratory tract inflammation, respiratory system irritation, mucous membrane and skin burns and reduction of the oxygen supply to the brain due to the chemical reaction of this product with water^11^. Effective, low-cost and natural methods can avoid the harmful health effects of ripening chemical inducers.

Merchant producers of the Borborema polo, Paraíba state, Brazil mature bananas with *Bowdichia virgilioides* Kunth (Fabaceae) leaves with results like those obtained with CaC_2_ but at lower cost. The ripening process with leaves of this plant includes the collection of *B. virgilioides* leaves at the coolest time of day, avoiding dew and excessive humidity during subsequent stages. The leaves of this plant are placed on the ground and the banana fruits are placed over them, but the proportion of leaves and fruits is not precise. The bananas and the leaves are then covered with plastic sheeting without air exchange between the external and internal environments. The tarp is left for 24 hours or longer depending on the fruit quantity. Masonry tanks of 1 m^2^ are also used with this method (personal farmer communication, 2017).

The sustainable management of *B. virgilioides* plants can facilitate the use of its leaves to induce banana ripening more economically. The objective was to test the empirical method of banana maturation using *B. virgilioides* leaves compared to the conventional method with CaC_2_.

## Results

Ethylene concentration was higher in atmosphere of the treatments with 4, 6, 8, and 10 g of leaves *B. virguloides*, followed by treatment with CaC_2_ in the laboratory ripening trial. In the field ripening trial, the ethylene concentration was higher in atmosphere of the treatment with leaves *B. virguloides*, followed by treatment with CaC_2_. Acetylene (C_2_H_2_) was only detected on treatment with CaC_2_ in the laboratory and field ripening trial. Respiratory rate, ascorbic acid content, malic acid, pH, and chlorophyll in ‘Pacovan’ bananas matured with *B. virgilioides* leaves and CaC_2_ do not differed (p < 0.05) in the laboratory (6, 8, and 10 g) and field ripening trial. The respiratory rate and ascorbic acid content of matured ‘Pacovan’ bananas was higher with *B. virgilioides* leaves and CaC_2_ than control in the laboratory (6, 8, and 10 g) and field ripening trial. The malic acid, pH, and total chlorophyll concentration of ripened ‘Pacovan’ bananas of *B. virgilioides* leaves and CaC_2_ was lower than control in the laboratory (6, 8, and 10 g) and field ripening trial (Table 1).

**Table 1:** Ethylene = C_2_H_4_ (ppm), Acetylene = C_2_H_2_ (ppm), Respiratory rate = RR (mg of CO_2_ Kg^−1^ h^−1^), ascorbic acid = AsA (mg 100 g^−1^), malic acid = MA (%), pH, and chlorophyll = Chlo (mg 100 g^−1^) in bananas matured with 2, 4, 6, 8 e 10 g of *Bowdichia virgilioides* leaves (T1, T2, T3, T4 and T5), carbide (g 50 Kg^−1^ carbide) (T6) and, uncoated with plastic film (T7) fruits covered with plastic film (T8) in the laboratory ripening trial and LBv = *B*.

Treat.	C_2_H_4_	C_2_H_2_	RR	AsA	MA	pH	Chlo
	Laboratory ripening trial						
T1	27.9 ± 2.1c	nd	17.6 ± 1.6b	16.1 ± 2.1b	45.0 ± 2.5a	5.6 ± 0.2a	10.3 ± 0.7a
T2	66.7 ± 4.9a	nd	48.6 ± 2.1a	35.3 ± 3.6a	38.8 ± 2.8a	4.9 ± 0.2b	10.8 ± 0.9a
T3	69.7 ± 5.8a	nd	52.4 ± 3.0a	40.9 ± 3.0a	33.7 ± 2.1b	5.0 ± 0.3b	1.2 ± 0.1b
T4	68.4 ± 6.4a	nd	54.6 ± 1.9a	33.8 ± 2.9a	32.6 ± 3.0b	4.6 ± 0.5b	1.2 ± 0.1b
T5	71.9 ± 7.4a	nd	59.7 ± 3.1a	42.6 ± 3.1a	35.3 ± 3.0b	4.5 ± 0.1b	1.0 ± 0.1b
T6	32.7 ± 3.5b	387.2 ± 12.2	45.3 ± 3.6a	45.7 ± 2.6a	38.0 ± 2.9b	4.5 ± 0.1b	2.3 ± 0.1b
T7	—	—	18.3 ± 1.0b	12.9 ± 0.9b	42.1 ± 3.0a	5.5 ± 0.1a	11.1 ± 0.1a
T8	19,8 ± 1,4d	nd	17.4 ± 0.9b	13.2 ± 0.6b	42.3 ± 2.4a	5.9 ± 0.1a	10.5 ± 0.1a
**CV %**	11.78	10.1	13.12	17.98	8.43	4.37	3.14
**Field ripening trial**							
LBv	65.6 ± 5.0a	nd	76.4 ± 9.2a	44.7 ± 4.1a	35.2 ± 3.9b	4.5 ± 0.1b	1,9 ± 0.1b
CaC_2_	61.9 ± 5.2a	317.1 ± 22.2	85.4 ± 9.8a	42.9 ± 5.1a	31.4 ± 4.9b	4,5 ± 0.1b	2.1 ± 0.1b
Control	34.1 ± 2.2b	nd	54.1 ± 8.7b	16.4 ± 3.1b	51.7 ± 7.9a	5.1 ± 0.1a	11.4 ± 0.5a
**CV (%)**	10.12	9.7	12.14	16.15	9.12	4.87	4.10

*virgilioides* leaves and CaC_2_=calcium carbide in the field laboratory ripening trial.

nd = not detected. *Means followed by the same letter per column do not differ by Tukey Test at 5% probability.

**Figure 1:**
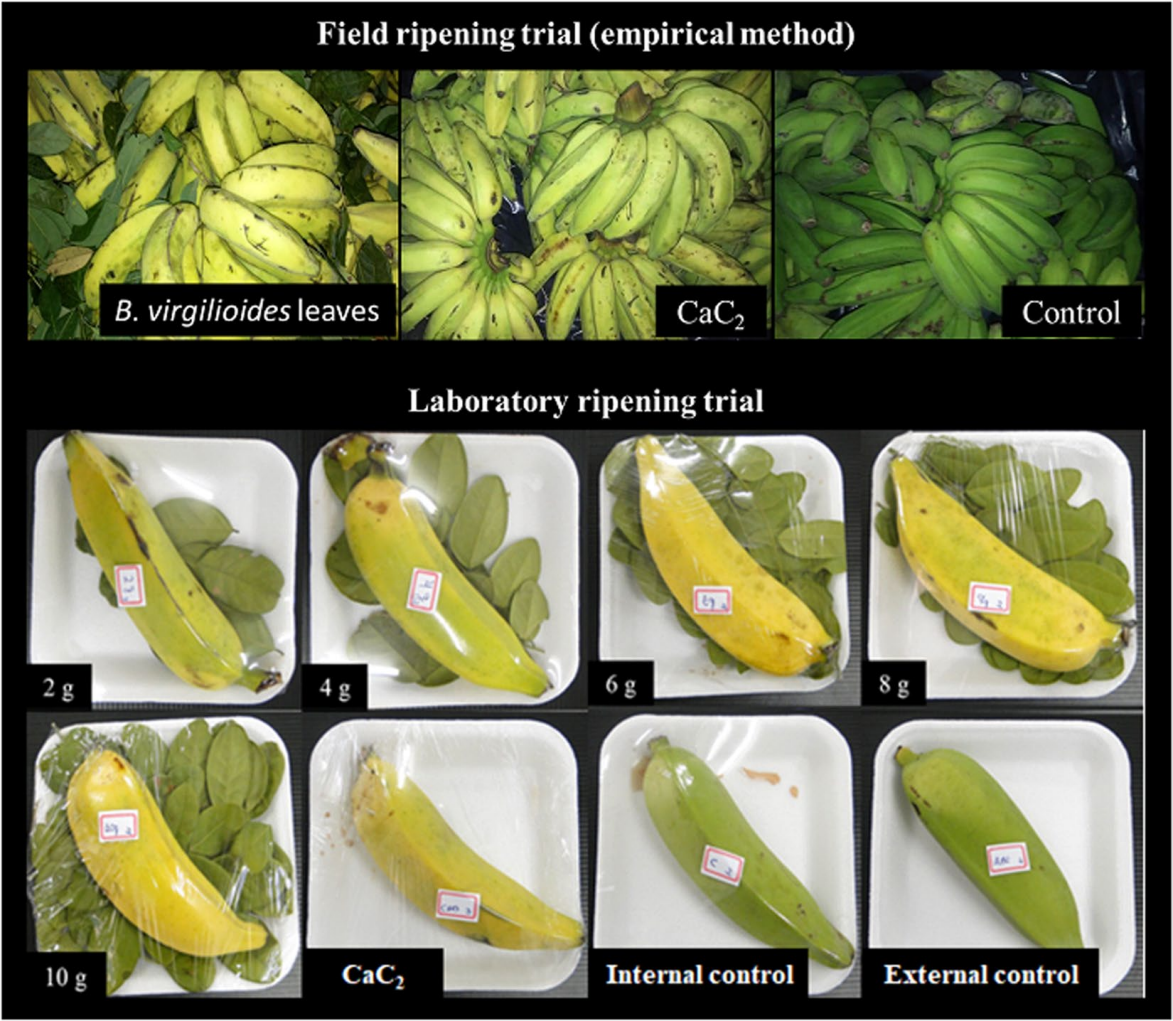
General appearance of bananas ripened with *Bowdichia virgilioides* leaves and carbide.

The ‘Pacovan’ banana peel ripened with *B. virgilioides* leaves and CaC_2_ changed color than control in laboratory (6, 8, and 10 g) and field ripening trial (Fig. 1).

## Discussion

The highest ethylene concentrations in the treatments with 4, 6, 8, and 10 g of *B. virgilioides* leaves are due to a cumulative effect of the gas produced by fruits and leaves. Ethylene concentration increases under atmospheric conditions modified by gas exchange limitation and autocatalysis^17^. The ethylene detected in the treatment with CaC_2_ was the autocatalytic produced by the banana fruits and induced by the calcium carbide. CaC_2_ may increase respiration rate, ethylene autocatalytic, chlorophyll degradation, carotenoid synthesis, starch conversion to sugar, increased activity of cell wall enzymes degradation, color change, texture, fruit aroma, and taste^18^. Acetylene is an ethylene analog used to initiate fruit ripening^19^. However, acetylene has lower biological activity than ethylene and higher concentrations for the same exposure period and for the same responses are needed^20^. In bananas, 0.01 ml L^−1^ of ethylene at 18 °C for 24 h began to ripen, while 1.0 ml L^−1^ of acetylene was required for a similar effect in several Florida hose cultivars^19^. The C_2_H_2_ was only detected on treatment with CaC_2_ due to the presence of this compound which is industrially produced and only releases C_2_H_2_ when reacted with water^21^.

The increase in the respiratory rate of ‘Pacovan’ bananas ripened with 6, 8, and 10 g of *B. virgilioides* leaves and CaC_2_ is due to the climacteric induction of respiration by the ethylene and acetylene emanated by *B. virgilioides* leaves and CaC_2_, respectively. Phosphofructokinase activity, which regulates this pathway^13^, produces energy (ATP) from starch degradation and hexose oxidation resulting in climacteric respiration^22^. In addition, the fruit exposure to ethylene and acetylene produced by *B. virgilioides* leaves and CaC_2_, respectively, may have increased the activity of the enzymes synthase and oxidase of ACC^23^ inducing climacteric respiration and accelerating maturation.

The highest ascorbic acid content (AsA) in bananas ripened with 6, 8, and 10 g of *B. virgilioides* leaves and CaC_2_ is due to the higher demand for AsA^24,25^ in these fruits, presumably by the most oxidized redox cell state^26,27^. The early fruit ripen, induced by ethylene and acetylene, produces reactive oxygen species^28^ increasing the demand for AsA reacting with superoxide, hydroxyl and peroxyl radicals, hydrogen peroxide, hypochlorite and singlet oxygen^29^. However, the biosynthesis of AsA is an antioxidant response by the D-glucosone, D-galacturonate, myo-inositol and D-mannose/L-galactose^30–32^. The AsA accumulation in these fruits may also be associated with the low oxidation of this pH-dependent molecule, with maximum at pH 5 and 11.5, being faster in alkaline media^33^ pathways but not necessarily related to its accumulation^34^.

The reduction of ripen ‘Pacovan’ banana acidity (malic acid) with 6, 8, and 10 g of *B. virgilioides* leaves and CaC_2_ is due to the oxidation of organic acids during fruit ripening by the increase in tricarboxylic acid cycle activity^35^. These acids were more rapidly and extensively degraded during the climacteric respiration^36^ induced by ethylene and acetylene, emanating from the *B. virgilioides* leaves and CaC_2_, respectively.

The pH reduction in ‘Pacovan’ bananas can be explained by the increase in the ascorbic acid content exceeding the titratable acidity reduction in the fruits matured with 6, 8, and 10 g of *B. virgilioides* leaves and CaC_2_. The AsA accumulation reduced the pH of these fruits due to the acidic character of this molecule attributed to the enodiol group (-HOC=COH-). The hydrogens of the enodiol group can dissociate, resulting in the strong ascorbic acid acidity and therefore are potential reducing agents^27^.

The lowest concentration of total chlorophyll in the ‘Pacovan’ banana peel ripe fruit induced with 6, 8 and 10 g of *B. virgilioides* leaves is due to the structural decomposition of chlorophyll by chlorophyllases, stimulated by ethylene^37^ and acetylene emanated from leaves and CaC_2_, respectively. The increase in the activity of these enzymes in these treatments coincides with the climacteric increase in fruit respiration^38^, which was also induced by ethylene and acetylene. Ethylene and acetylene its analogues accelerate the chlorophyll losses^39^ and regulates the yellowing of banana peels^40^. The drop of total chlorophyll concentration in banana fruits induced by CaC_2_ was similar due lower biological activity than ethylene and higher concentrations for the same exposure period and for the same responses are needed^20,41^.

## Conclusion

The method used by Borborema producers in the Paraíba state, Brazil to ripen ‘Pacovan’ bananas with *Bowdichia virgilioides* leaves is safer and has the same results than those obtained with carbide.

## Material and Methods

### Location and raw material

Banana bunches of the ‘Pacovan’ variety, from agroecological production, were harvested in the early hours in the morning. Banana fruits were selected and standardized according to size, absence of physiological defects and infections, at the maturation stage 3 with yellowish green color^42^. Part of the harvested fruits were transported to the laboratory and part remained in the field. Banana ripening was evaluated with *B. virgilioides* leaves (harvested according to the producers orientation) and calcium carbide (CaC_2_) in the field and laboratory.

### Laboratory ripening trial

‘Pacovan’ banana bites were scrapped with a stainless-steel knife and the fruits were, individualized in trays of expanded polystyrene for 30 min, to reduce the ethylene effect produced in their wound. The treatments were 2.0 g of *B. virgilioide*s leaves + plastic film coating (T1); 4.0 g of *B. virgilioides* leaves + plastic film coating (T2); 6.0 g of *B. virgilioides* leaves + plastic film coating (T3); 8.0 g of *B. virgilioides* leaves + plastic film coating (T4); 10.0 g of *B. virgilioides* leaves + plastic film coating (T5); CaC_2_ (g 50 kg^−1^) + coating with plastic film (T6); internal control (only coated with plastic film) (T7) and external control (without coating with plastic film) (T8) per banana. Treatments were stored at 27 ± 2 °C and relative humidity of 87 ± 5%. The fruits remained under these conditions for 48 hours and were then evaluated. The experiment was developed in triplicate.

### Field ripening trial (empirical method)

Two and a half kilograms of *Bowdichia virgilioides* leaves were placed covering the entire soil and 100 kg of banana fruits are placed over them. The bananas and the leaves were then covered with tarp without air exchange between the external and internal environments. The tarp was left for 24 hours (personal farmer communication, 2017). The same procedure was performed by replacing the leaves with CaC_2_ (0,5 g kg^−1^ of fruit). The control consisted only of the fruits covered by the tarp.

### Ethylene and acetylene quantification

Ten air samples were withdrawn with syringes from the atmosphere beneath the tarp. The syringes needle tip were sealed with rubber and immediately taken to the laboratory where were injected into a GC-14B (ShimadzuCrop Kyoto Japan), with Porapak-Q packaged column and flame ionization detector for ethylene and acetylene analysis.

### Respiratory rate

Banana fruits were placed in hermetically sealed containers with 10 mL of 0.5 N NaOH. The CO_2_, produced by the fruits, was measured by titration and after 24 h, the NaOH was titrated with 1 N HCl with results expressed as mg of CO_2_ kg^−1^ h^−1^. The respiratory rate was estimated by the equation: mgCO_2_ Kg^−1^ fresh matter = (B − L) × C/MF where B = volume in mL spent for titration of the “control” (container without fruit, only with NaOH); L = volume spent to neutralize NaOH; C = correction factor (0.98); MF = fresh fruit mass. The hourly respiratory rate was determined with the formula mg CO_2_ Kg^−1^ h^−1^ = mg CO_2_ g^−1^ fresh matter × 1000/IT; IT = time interval between titrations (24 h).

### Ascorbic acid

ascorbic acid content was determined by titration with a 0.02% 2,6-diclophenol-indophenol (DFI) indicator solution in 5.0 g of fresh banana mass diluted in 30 ml of oxalic acid at 0.5%^43^.

### Titratable acidity

The malic acid content (acidity) of the banana was determined by titrametry in five-gram pulp samples of the fruit diluted in 50 mL of distilled water. Then, 4–5 phenolphthalein indicator droplets were added and titration performed with 0.1 N NaOH^44^.

### pH

The pH was determined in digital bench pH meter in samples, 30 min after the dilution of five grams of banana pulp in 50 ml of distilled water.

### Total chlorophyll

Two grams of the banana peel were macerated with 7 mL of 80% (v/v) acetone and the extract filtered and the volume then filled with 80% (v/v) acetone. The absorbance was read at wavelengths at 646.8 and 663.2 nm, and calculated by the equation: total chlorophyll (T) = 7.15 × A_663.2_ + 18.71 × A_646.8_ ^45^.

### Experimental design and data analysis

The experiment was carried out in a completely randomized design with eight treatments and four replications. Each experimental unit had one banana per tray. The results were submitted to variance analysis by the F test and the means compared by the Tukey test (P < 0.05) with the program Assistat version 7.7.
